# Diversity, Equity and Inclusion in an Age-Friendly Health System

**DOI:** 10.1093/geroni/igab046.3382

**Published:** 2021-12-17

**Authors:** Elizabeth Eckstrom, Emily Morgan, Bryanna De Lima, Anna Pleet

**Affiliations:** 1 Oregon Health & Science University, Portland, Oregon, United States; 2 Oregon Health and Science University, Portland, Oregon, United States; 3 Vita-Salute San Raffaele University, Milan, Lombardia, Italy

## Abstract

The Age-Friendly Health Systems (AFHS) initiative uses a 4Ms framework to encourage patient-centered care for older adults. Many health systems have implemented the core elements of AFHS – What Matters, Mentation (Cognition and Depression), Medications, and Mobility – with the goal to uniformly apply these elements to all patients 65 years and older. However, equity in AFHS delivery has not yet been examined. Five diversity, equity, and inclusion (DEI) factors, including gender, race, ethnicity, preferred language and MyChart activation, were cross-sectionally analyzed against the 4Ms framework for patients seen (in person or virtual visit) in an academic internal medicine clinic between April 2020 and April 2021 (N= 3370) using two-way contingency tables. Preferred language, gender, and MyChart activation yielded significant pairings with the 4M metrics. For the AFHS What Matters metric, females were 1.10 times more likely than males and English-speaking patients were 1.67 times more likely than non-English speaking patients to receive advance care planning (p <0.01). Females and patients with MyChart activation were about 2.0 times more likely to have a high-risk medication on their medication list compared to males and patients without MyChart activation (p <0.01). MyChart activation was also significantly associated with cognitive screening. Patients with MyChart activation were 1.09 times more likely than patients without MyChart activation to get cognitive screening (p <0.001). This study, the first to incorporate demographic data, into AFHS quality measures, suggest a need to develop best practices for equitable Age-Friendly care at the clinical team and the institutional policy level.

